# Implementing nudges for suicide prevention in real-world environments: project INSPIRE study protocol

**DOI:** 10.1186/s40814-020-00686-y

**Published:** 2020-09-26

**Authors:** Molly Davis, Courtney Benjamin Wolk, Shari Jager-Hyman, Rinad S. Beidas, Jami F. Young, Jennifer A. Mautone, Alison M. Buttenheim, David S. Mandell, Kevin G. Volpp, Katherine Wislocki, Anne Futterer, Darby Marx, E. L. Dieckmeyer, Emily M. Becker-Haimes

**Affiliations:** 1 Department of Psychiatry, University of Pennsylvania Perelman School of Medicine, Philadelphia, PA USA; 2 Penn Implementation Science Center at the Leonard Davis Institute of Health Economics (PISCE@LDI), University of Pennsylvania, Philadelphia, PA USA; 3 Leonard Davis Institute of Health Economics, University of Pennsylvania, Philadelphia, PA USA; 4 Department of Medical Ethics and Health Policy, Perelman School of Medicine, University of Pennsylvania, Philadelphia, PA USA; 5 Department of Medicine, Perelman School of Medicine, University of Pennsylvania, Philadelphia, PA USA; 6 Department of Child and Adolescent Psychiatry and Behavioral Sciences, Children’s Hospital of Philadelphia, and PolicyLab, Children’s Hospital of Philadelphia, Philadelphia, PA USA; 7 Center for Health Incentives and Behavioral Economics, Perelman School of Medicine, University of Pennsylvania, Philadelphia, PA USA; 8 Department of Family and Community Health, School of Nursing, University of Pennsylvania, Philadelphia, PA USA; 9 Department of Health Care Management, The Wharton School, University of Pennsylvania, Philadelphia, PA USA; 10 Penn Medicine Center for Health Care Innovation, University of Pennsylvania, Philadelphia, PA USA; 11 Jefferson College of Life Sciences, Thomas Jefferson University, University of Pennsylvania, Philadelphia, PA USA

**Keywords:** Suicide, Prevention, Implementation science, Primary care, Mental health

## Abstract

**Background:**

Suicide is a global health issue. There are a number of evidence-based practices for suicide screening, assessment, and intervention that are not routinely deployed in usual care settings. The goal of this study is to develop and test implementation strategies to facilitate evidence-based suicide screening, assessment, and intervention in two settings where individuals at risk for suicide are especially likely to present: primary care and specialty mental health care. We will leverage methods from behavioral economics, which involves understanding the many factors that influence human decision making, to inform strategy development.

**Methods:**

We will identify key mechanisms that limit implementation of evidence-based suicide screening, assessment, and intervention practices in primary care and specialty mental health through contextual inquiry involving behavioral health and primary care clinicians. Second, we will use contextual inquiry results to systematically design a menu of behavioral economics-informed implementation strategies that cut across settings, in collaboration with an advisory board composed of key stakeholders (i.e., behavioral economists, clinicians, implementation scientists, and suicide prevention experts). Finally, we will conduct rapid-cycle trials to test and refine the menu of implementation strategies. Primary outcomes include clinician-reported feasibility and acceptability of the implementation strategies.

**Discussion:**

Findings will elucidate ways to address common and unique barriers to evidence-based suicide screening, assessment, and intervention practices in primary care and specialty mental health care. Results will yield refined, pragmatically tested strategies that can inform larger confirmatory trials to combat the growing public health crisis of suicide.

## Background

Suicide is a critical health issue around the world; about 800,000 people die by suicide each year, which translates to one death every 40 s [1]. In fact, suicide is now the second leading cause of death among individuals 15–29 years of age globally [1]. In the United States, suicide rates across the lifespan increased by 35% from 1999 to 2018 [2], highlighting the need for widespread use of effective screening, assessment, and intervention tools to reduce the number of lives lost to suicide. While evidence-based suicide screening, assessment, and intervention (referred to hereafter as SSAI) practices exist to mitigate suicide risk [3, 4], implementation of these practices often is inadequate [5, 6]. There is a dearth of knowledge about why evidence-based practices (EBPs) for SSAI remain underused and how to optimize implementation to increase the number of lives saved. The limited research in this area indicates that clinicians routinely report misconceptions and anxiety about screening for suicide [7, 8]. Research from other areas of medicine suggests that clinicians—like all humans—employ heuristic decision-making and are subject to common behavioral barriers [9]. In the case of clinicians, these behavioral barriers and heuristics may interfere with the uptake of EBPs, but can also be effectively targeted via implementation strategies that seek to improve EBP use [9]. However, the specific behavioral barriers that interfere with the implementation of SSAI EBPs across healthcare settings, as well as the most promising strategies to address these barriers, are unknown.

This exploratory project will (a) identify key behavioral barriers that may limit the implementation of SSAI EBPs and (b) develop and test behavioral economics-informed implementation strategies to target those barriers. To maximize the impact of this work, we will study two settings particularly critical for suicide prevention: primary care and specialty mental health.

Developing cross-setting implementation strategies that can be deployed across the multiple setting types where individuals at risk for suicide commonly present has the potential to lead to more efficient implementation. Most people who die by suicide interact with a primary care clinician in the prior year and close to one-third of individuals who die by suicide are seen by an outpatient mental health provider in the year before their death [10]. Thus, concomitant attention to supporting SSAI EBPs in both settings is critical to meeting the needs of those at risk for suicide. Identifying cross-setting implementation strategies, as well as those that address barriers unique to each setting, will help ensure strategies match the needs of at-risk patients seen in primary care and specialty mental health. The current study seeks to generate implementation strategies that can be more efficiently deployed across a range of healthcare settings; this approach can help advance the field of implementation science.

### Current implementation of suicide prevention EBPs

Several effective screening [11, 12], assessment [3, 13], and intervention [4] approaches for addressing suicide risk have been deployed across a range of healthcare settings [14, 15]. Moreover, a number of national guidelines have been put forth regarding suicide screening across settings. For instance, physician organizations such as the American Academy of Pediatrics recommend that for patients presenting with behavioral health difficulties, clinicians should screen for suicide risk [16]. Although universal screening for suicide risk (i.e., screening all patients regardless of the primary presenting problem) is not required in primary care, the U.S. Preventive Services Task Force recommends (B grade) universal depression screening for adolescents [17] and adults [18] in primary care. Thus, as more providers increasingly screen for depression in line with the aforementioned guidelines, there is likely to be a concomitant rise in the identification of individuals at risk for suicide across a number of healthcare settings.

### Potential barriers to implementation

The extant literature describes key factors that may impede consistent use of SSAI EBPs, including gaps in clinician training and knowledge, time constraints, beliefs that suicide screening increases risk, and assuming that SSAI is someone else’s job [7, 8, 19]. However, most prior research on barriers to SSAI EBP implementation focuses on a single EBP or setting [19] and there are significant gaps in our understanding of the mechanisms that lead to poor implementation of SSAI EBPs. More work is needed to understand how these mechanisms operate to identify optimal targets for implementation strategies. In addition, little is known about how barriers may overlap and vary as a function of whether the EBP of interest relates to suicide screening, assessment, or intervention and whether these barriers are similar or distinct across different health settings. For example, clinician “forgetting” may be an important barrier to the implementation of suicide screening, whereas clinician beliefs about whether their professional role includes suicide intervention might be a more important factor in whether a clinician engages in any follow-up intervention to address suicide risk. Further distinguishing barriers that cut across settings and EBPs from those that are unique to a specific context and/or EBP can inform the extent to which implementation strategy development can be expedited to address common barriers. This can have life-saving implications and can shorten the research-to-practice gap.

### Leveraging insights from behavioral economics

Given that clinicians’ beliefs are commonly cited as barriers to SSAI practices [7], behavioral economics (BE) strategies may be particularly well-suited for enhancing the implementation of EBPs for suicide prevention across settings. BE comprises a set of theories and frameworks that recognize that humans demonstrate predictably bounded rationality, meaning they often make decisions heuristically, based on incomplete information and without exhaustive analysis of all costs and benefits [20]. As an example, prospect theory [21] describes how people make decisions based on the perceived likelihood of gains and losses. Additionally, BE principles suggest people have limited self-control, limited attention and cognitive processing power, are present- rather than future-oriented, and are strongly influenced by social norms [20–24].

To date, a number of behavior change theories such as the theory of planned behavior [25] and self-determination theory [26] have been used to guide implementation research [27, 28]. BE offers unique insights for the design of implementation strategies that target human decision-making constraints (i.e., cognitive heuristics) across settings. BE approaches have yielded notable effects in changing clinician practices in other health domains [29–32]. For instance, changing the default options in the electronic health record (EHR) has been found to increase the prescribing of generic medications over pricier, brand-name medicines [29]. Nonetheless, taxonomies of implementation strategies, such as the Expert Recommendations for Implementation Change (ERIC [33]), do not include consideration of BE principles for targeting behavioral barriers specifically nor do they address potential mechanisms of implementation more broadly. This omission means that implementation strategies are seldom informed by principles of BE [34]. In particular, innovative BE-informed implementation strategies for preventing suicide via EBPs remain untapped. Subtle but powerful “nudges” that change the choice architecture (i.e., the way choices are presented) in which clinicians make screening and treatment decisions [32] are one example of a potential BE-informed implementation strategy for increasing SSAI EBP use.

### Leveraging methods from innovation science and industry

Rapid cycle approaches (RCAs) are derived from innovation science to “fail fast and learn quickly” in the search for effective implementation strategies and may be well-suited for healthcare settings [35, 36]. RCAs represent a set of methods to develop and refine an innovation without investing the resources required to develop a full-scale version [37]. RCAs leverage observation and iterative testing of innovations integrated within clinic operations to learn how to design innovations (in this case, implementation strategies) to fit a clinic workflow in an efficient, cost-effective, and reliable way. The typical development and refinement process involves several cycles of concept definition, implementation of a skeletal system, evaluation, and concept refinement. Given the cost of developing fully functioning implementation strategies, RCAs are ideal approaches through which to prototype and optimize strategies. RCAs allow researchers to test the “assumptions” underlying why individuals may not use a particular EBP. By rapidly proving or disproving the assumptions underlying the barriers to SSAI EBP use (i.e., the mechanisms of implementation), we can ensure that the final implementation strategies are designed for the correct targets. For example, participants may highlight as a barrier that safety planning materials are difficult to access in the EHR. There are assumptions underlying this barrier, such as that clinicians have the requisite knowledge to engage in safety planning but lack the means to do so. RCAs are designed to test such assumptions to inform refinement or design of new implementation strategies. A strategy such as developing and testing a more user-friendly approach to accessing the safety planning template in the EHR would test the assumption that the primary barrier is the lack of access to safety planning materials. If this then leads to increases in safety planning, we can be more confident in the accuracy of this assumption and the possible utility of this strategy as an implementation strategy at scale. If safety planning does not increase, we would then need to test possible alternative explanations (e.g., that clinicians do not have the confidence to safety plan). The outcome of RCAs is a set of implementation strategies that are ready to be tested in larger confirmatory trials since there is preliminary support suggesting the implementation strategy engages the correct mechanism.

### Aims

This project will merge insights from BE and implementation science along with methods from innovation science to identify key barriers to SSAI and to generate implementation strategies to increase the use of SSAI EBPs that can be applied broadly across different healthcare settings. This study comprises a project conducted as part of a National Institute of Mental Health-funded P50 Advanced Laboratories for Accelerating the Reach and Impact of Treatments for Youth and Adults with Mental Illness (ALACRITY) grant (supplement grant number: 3P50MH113840-03S1; PIs: Beidas, Mandell, & Buttenheim/Volpp), which aims to accelerate the pace at which effective treatments for psychiatric disorders are deployed in community practice by integrating principles of implementation science, BE, and participatory design [38]. In partnership with a team of key stakeholders, including clinicians, setting leaders, behavioral economists, implementation scientists, and suicide experts, we propose to:

- Aim 1: Identify barriers and facilitators to SSAI implementation. We will apply established approaches to contextual inquiry (surveys and interviews [39, 40]), in addition to the assessment of fidelity to SSAI EBPs, to elicit key barriers that limit the implementation of suicide prevention EBPs in each setting and map identified barriers to established BE frameworks (e.g., the EAST framework, described below [41]). The qualitative interview will be guided by behavioral science tenets outlined by Potthoff and colleagues [42] that capture the volitional and implicit aspects (i.e., dual-process model) of clinician behavior as well as the competing goals clinicians face (i.e., multiple behavior approach). A questionnaire will be administered to tap attitudes that may serve as barriers to safety planning [43].
  - Rationale. Traditional approaches have leaned heavily on surveys and qualitative interviews to glean barriers to implementation. Although surveys and interviews can be informative, clinicians may not always be aware of the barriers to implementation, particularly those driven by their own heuristics. Thus, we will complement our contextual inquiry with baseline fidelity monitoring of current SSAI practices, using chart-stimulated recall (i.e., brief, structured interviews during which the clinician reviews patients’ charts to aid recall of specific encounters [44–48]). This approach has been used as a fidelity measure in other research [49] and will yield a rich understanding of current SSAI practices and barriers to implementing such practices. As described above, BE will be an important lens through which such barriers are interpreted.
- Aim 2: Match implementation strategies to identified barriers**.** Insights gleaned from Aim 1 will be leveraged to design a menu of BE-informed implementation strategies, with input from key stakeholders that cut across settings.
  - Rationale. The goal is for these strategies to be useful in both primary care and specialty mental health settings. To achieve this goal, it is essential for key stakeholders from various settings to be involved in generating, evaluating, and refining potential strategies.
- Aim 3: Prototype implementation strategies using RCAs. The final study aim is to evaluate the feasibility and acceptability of the BE-informed implementation strategies using RCAs.
  - Rationale. In addition to the feasibility and acceptability of the implementation strategies, we will also assess fidelity to SSAI procedures before, during, and after each rapid cycle phase using chart-stimulated recall. As described above, chart-stimulated recall is a method for indexing clinician fidelity [49] and will therefore provide important, minimally intrusive information on clinicians’ adherence to SSAI procedures in each practice before, during, and after rapid cycle trials.

## Method

### Settings

This project will be conducted in partnership with primary care clinics (*n* = 4; two clinics with a pediatric focus, one internal medicine practice, and one family medicine site) and specialty mental health programs (*n* = 2). Although some SSAI EBPs are either mandated or recommended in each of these settings, each setting is at a different stage of implementation of SSAI EBPs. Sites were intentionally selected for the present study to include a combination of primary care and specialty mental health settings that represent variability in current SSAI practices. For example, in the primary care networks from which specific clinics have been sampled, leadership has implemented guideline-concordant depression screening for adolescents and adults. When suicidal ideation is endorsed, additional follow up by behavioral health or primary care clinicians is recommended; however, exact protocols and procedures vary by practice. In the specialty mental health programs, insurance regulations require documentation of the presence or absence of suicidal ideation in each clinical encounter, but do not require clinicians to use routine screening tools across clinical encounters; administration of the Columbia-Suicide Severity Rating Scale [3] to assess suicidality is encouraged but not mandatory. When suicide risk is present for patients seen in the specialty mental health programs, clinicians are required to document a safety plan in the patient’s chart. However, clear guidelines as to how SSAI should be conducted are lacking and the rates at which SSAI EBPs are used in this specialty mental health setting are unknown.

The relevant Institutional Review Boards (IRBs) have approved this study and all ethical guidelines will be followed. All research participants (i.e., behavioral health and primary care clinicians, clinic leaders) will provide written consent prior to participating. Given that this is a provider-facing study and all study activities will take place only with providers, patients will not be enrolled. Data will be de-identified to protect confidentiality. Data will be available to members of the research team for analyses. This report was prepared in accordance with Standard Protocol Items: Recommendations for Interventional Trials (SPIRIT) 2013 guidelines [50]. We have completed recruitment and data collection for Aim 1; data collection for Aim 3 has begun in specialty mental health but is not completed and has not yet commenced in primary care. No data has been analyzed for any of the primary study aims. We plan to work with practice leadership to adjust study planning as needed in light of COVID-19 social distancing requirements to ensure that all data collection and rollout of implementation strategies can be conducted remotely. We will also consult with the relevant IRBs to ensure the appropriate ethical approvals are obtained for changes made in partnership with stakeholders.

### Aim 1: identify barriers and facilitators to SSAI implementation

#### Sample

Our participants will include clinicians from primary care (*n* = 10; a combination of behavioral health and primary care clinicians) and specialty mental health (*n* = 10; behavioral health clinicians and case managers) sites. We will use purposive sampling to select clinicians for this aim. Clinic leadership will nominate clinicians whom they consider champions of SSAI (e.g., those who frequently screen for suicide risk) and those whose use of SSAI is indicative of typical provider practice so that we can learn from those who engage in ideal and usual practice. We will invite these individuals to participate.

#### Procedures

Each clinician will complete an individual, semi-structured interview, as described below, which will query about barriers and facilitators to SSAI implementation, in addition to a questionnaire about attitudes toward safety planning. At the end of the qualitative interview, clinicians will engage in chart-stimulated recall to provide information on their recent SSAI practices with patients.

#### Measures

##### Attitudes toward safety planning

The Clinician Attitudes Toward Safety Planning questionnaire [43] will assess clinicians’ attitudes toward safety planning. Reyes-Portillo et al. [43] reported that Cronbach’s alpha for the 12 attitude items on this measure was .83.

##### Qualitative interview on barriers and facilitators

Members of the research team will conduct theory-guided qualitative interviews adapted from Potthoff et al. [42]. Potthoff et al.’s [42] interview was developed to understand the implementation of type 2 diabetes self-management and captured the volitional and implicit aspects (i.e., dual-process model) of clinician behavior as well as the competing goals clinicians face (i.e., multiple behavior approach). The current interview will involve questions about clinicians’ SSAI practices, how they make decisions about escalating to a higher level of care (e.g., sending a patient to the emergency department), as well as a number of factors that may facilitate or impede implementation of suicide prevention practices (e.g., self-efficacy, competing demands, motivation).

##### Chart-stimulated recall

For the chart-stimulated recall (i.e., the fidelity assessment), a member of the research team will review the clinician’s caseload with them for a specified, recent clinic day. For each patient seen that day, the researcher will ask brief questions (no more than 5 min) related to the clinician’s suicide practices (e.g., Did you conduct a screen for suicide risk? How did you screen for risk?). Clinicians will provide yes/no responses to this fidelity assessment and will be able to provide a rationale (e.g., why they opted not to screen for suicide risk) as needed.

#### Analyses

##### Qualitative coding

Interviews will be digitally recorded and transcribed with analyses supported by the use of an NVivo database. Using an integrated approach [51] to codebook development, a priori codes will be developed using constructs from BE derived from the EAST framework (described below [41]) and additional codes will be added to the codebook by the research team following a close reading of the first five transcripts [52, 53]. This process will result in a structured codebook in which each code will be defined and decision rules for their application are included in the definition.

Briefly, the EAST framework asserts that to encourage behavior change and reduce people’s reliance on cognitive biases, the desired change should be Easy, Attractive, Social, and Timely. Specifically, the EAST principals are (a) Make it Easy to change behavior, by using strategies such as default options (e.g., auto-enrollment in 401 k), reducing the effort required to perform an activity, and simplifying messaging; (b) Make it Attractive, by using strategies such as attention-grabbing messaging and incentives; (c) Make it Social, by using strategies that demonstrate that most people engage in the desired behavior, harnessing the power of networks, and encouraging people to express commitment to others; and (d) Make it Timely, by using strategies such as the provision of prompts when people are most likely to be receptive, encouraging consideration of immediate costs and benefits, and engaging in planning for action. We will code for barriers and facilitators that make it easier or harder, more or less attractive, social, and timely to implement SSAI EBPs.

Using the NVivo qualitative data analysis software program, two members of the research team will then separately code a new sample of three transcripts and compare their application of the coding scheme to assess the reliability and robustness of the coding scheme. Disagreements in coding will be resolved through discussion and the codebook will be refined and applied to all transcripts. Coders will be expected to reach and maintain interrater reliability at Cohen’s kappa ≥.8. Reliability will be monitored through biweekly coding meetings. If reliability drops below the set kappa value of .8, additional training, supervision, and consensus meetings with coders will be conducted to improve reliability.

Themes that generalize across sites, as well as those specific to a given setting (i.e., primary care or specialty mental health) will be summarized.

##### Attitudes toward safety planning

Descriptive statistics will yield information on clinician attitudes toward safety planning.

##### Chart-stimulated recall

Since each setting has certain institutional expectations regarding SSAI practices, those expectations will be used to determine fidelity benchmarks. For instance, if a given site has an expectation that screening for suicide risk should occur at all visits for patients above a certain age, the number of patients in that age range who were screened out of the total number of patients seen in that age range will be used to calculate fidelity (all based on chart-stimulated recall). Responses to chart-stimulated recall items will be analyzed separately for each SSAI practice (e.g., screening separately from intervention).

Findings from Aim 1 will be synthesized to identify barriers and facilitators impacting the implementation of SSAI EBPs that will serve as inputs to implementation strategy design in Aim 2.

### Aim 2: Match implementation strategies to identified barriers

#### Sample

Participants will include an advisory board of expert stakeholders, including behavioral economists, clinicians, implementation scientists, and suicide prevention experts. This group will develop prototypes of implementation strategies that can help increase the use of SSAI EBPs by targeting the barriers identified in Aim 1.

#### Procedures

With guidance from one of the co-authors who is a design strategy consultant, the investigative team will engage in an assumption mapping exercise drawn from innovation [54] to first identify assumptions relevant to SSAI EBP implementation (e.g., “organizational leadership has established clear policies on how suicidal risk should be handled at their site”) and determine the degree of certainty and risk associated with each assumption (e.g., by asking questions such as “How certain are we that this assumption is true?” and “If we are wrong, how problematic would that be to successful implementation?”).

Qualitative interviews and surveys from Aim 1 will form the basis for the assumption mapping exercise. Members of the research team will use barriers to SSAI identified through Aim 1 data collection to engage in a guided exercise led by the design consultant to create a comprehensive list of assumptions underlying identified barriers to the use of SSAI EBPs. Aim 1 data will inform team ratings of “how likely” it is that each assumption is true and “how problematic” it would be if each assumption were false. For example, if an identified barrier is that a lack of clear protocols inhibits SSAI implementation, an assumption underlying that barrier might be that enhancing protocols would promote the use of SSAI EBP practices. As described further below, we will then design and roll out an implementation strategy (i.e., a method for overcoming barriers to incorporating a clinical practice into routine healthcare delivery [55]) during the Aim 3 RCAs to test the assumptions generated.

The assumption mapping exercise will produce a matrix of assumptions falling into four categories: “likely true, not problematic if false,” “likely true, problematic if false,” “uncertain if true, not problematic if false,” and “uncertain if true, problematic if false.” Assumptions rated as “uncertain if true, problematic if false” constitute the “highest risk” assumptions. This category represents the highest level of risk because there is both uncertainty as to whether the assumption is true and, if the assumption is false, targeting that barrier with an implementation strategy is unlikely to lead to successful EBP implementation. These high-risk assumptions are therefore most crucial to test in using RCAs to optimize the likelihood that the implementation strategies identified for testing in confirmatory trials target the correct mechanisms. For example, one “high-risk” assumption might be “Clinicians know how to implement SSAIs, but lack of time makes it difficult for them to do so.” If false, this could mean that limited SSAI limitation is due to other factors, such as clinicians being asked to use interventions that they are improperly trained to deliver. Thus, testing this assumption (e.g., by leading trainings or brief seminars and obtaining feedback about the extent to which the training contained new information) would be critical for determining whether the final set of implementation strategies to be tested in larger trials should address clinician knowledge.

We will design prototyped versions of implementation strategies that will test the highest risk assumptions through rapid prototyping using RCA methodology in Aim 3. The goal of these trials is not to test the effectiveness of the implementation strategy, but rather to test the underlying assumption and evaluate the acceptability and feasibility of the implementation strategy. The EAST framework will inform the design of the implementation strategy to test the underlying assumption. For instance, if an underlying assumption is that clinicians forget to screen, we would prototype an attractive, timely reminder for clinicians to screen. If, during prototyping, providers give feedback that they saw the reminder and found it feasible and acceptable but did not screen because they thought the patient was too young or low risk, we would learn our original assumption about “forgetting” was not correct. This would lead us to test both a new implementation strategy and, in turn, an alternative assumption (e.g., clinician beliefs about client appropriateness is a primary driver of the decision to screen). Alternatively, if the provider states that they never saw the reminder, that would suggest that the implementation strategy needs to be redesigned to serve its intended purpose.

Methods from innovation will be used as appropriate to develop and assess the prototyped implementation strategies, such as “fake back ends” and “vapor tests,” before larger, more comprehensive solutions are formulated and tested [37]. A fake back end is a temporary infrastructure that mimics a more resource-intensive strategy. For example, rather than programming a text-messaging platform that reminds clinicians to screen patients for suicide risk, a fake back end would entail a study team member manually texting reminders to clinicians to determine whether the system warrants the investment to automate it. A vapor test is a way to realistically assess demand for a given product or service to determine whether that product/service should be created. For example, a vapor test could be used to ask primary care clinicians to sign up to use an on-call service for access to a behavioral health clinician for consultation regarding suicide risk. Upon signing up for the service, the primary care clinician would receive a message that the service is “coming soon.”

Key stakeholders will provide feedback on the refined set of assumptions and initial prototyped implementation strategies. The approach used in the current study of applying established practices from innovation to generate solutions to issues in healthcare is consistent with suggestions in the extant literature [37], yet such approaches are scarcely applied in behavioral health domains.

The product will be a list of potential implementation strategies mapped to assumptions, which we will evaluate in Aim 3. Findings will also yield important insights on the feasibility and utility of using an assumption mapping approach to design tailored implementation strategies. See Table 1 for an example of hypothesized output; however, this is subject to change based upon what we learn in the first aim.

**Table 1:** Examples of applying tailored implementation strategies to target potential assumptions

Barrier identified via contextual inquiry	Underlying assumption	Behavioral insight	Implementation strategy to be tested	Plan for testing variations of the implementation Strategy	Sample questions for rapid prototyping evaluation
Clinicians report “forgetting” as a barrier to routine screening	Clinicians intend to screen and would do so more often if they remembered	Cognitive load	Clinicians nudged to screen via posters or text reminders prior to session times	Test different posters hanging in the hallway with various framed messages and in different locations; send text reminders before appointment times to nudge clinicians to screen	Did you notice the poster in the hallway? Did you notice the text message before your sessions? What was it like having a reminder to screen?
Clinicians do not perceive that others in their organization routinely engage in brief intervention to address suicide risk	Clinicians do not believe that brief interventions for suicide risk are the norm in their practice and therefore use these interventions infrequently	Social norms	Weekly leaderboard of clinicians engaging in safety planning for suicide prevention with clients identified to be at suicide risk	Vary timing of the email, who sends it (clinic leader vs. research team), and presentation of the results	Did you open the email? What were your thoughts when you saw the leaderboard?

### Aim 3: prototype implementation strategies using RCAs

#### Sample

Participants in Aim 3 will include behavioral health clinicians and physicians (*n* = 10) in primary care practices, and behavioral health clinicians (*n* = 10) in specialty mental health programs. Aim 3 participants may overlap with those in Aim 1 but new clinicians will also be invited to participate in Aim 3.

#### Procedures

We will start by deploying an implementation strategy that addresses barriers and tests assumptions in small steps to build up to a larger strategy that can be tested in future studies. In that way, we will be able to “rapidly test critical assumptions in context” [37]. For a given rapid cycle period, our research team will embed themselves in the sites to engage in brief surveys and interviews regarding the rollout of each implementation strategy (note: we will work closely with stakeholders to ensure that all processes comply with COVID-19 restrictions). After testing a given implementation strategy, the team will debrief and decide whether to refine or adjust strategies based on the following criteria: (a) were enough trials completed to gain insight that consistently validates or invalidates the assumption?; (b) were a breadth of relevant stakeholders or circumstances included?; (c) were there obvious failures (e.g., did stakeholders voice serious concern)?; and (d) was the SSAI metric in question affected by the strategy?. We will then systematically test other strategies or refine existing strategies to improve their integration into clinic workflow. We anticipate that each phase will take 1–3 weeks and that we will conduct up to five rapid cycle trials. Of note, rapid prototyping is designed to uncover additional barriers and facilitators in order to optimize implementation strategies.

#### Measures

After each discrete rapid cycle phase, we will quantitatively assess the feasibility and acceptability of the implementation strategies from the perspective of clinicians via two brief (4 items each) questionnaires: Acceptability of Intervention Measure and Feasibility of Intervention Measure [56]. These questionnaires have demonstrated reliability and validity (e.g., internal consistency, test-retest reliability, content validity [56]. We also will collect qualitative data by directly asking clinicians, staff, and clinic leaders about their experience with each strategy. To obtain a preliminary estimate of fidelity to SSAI procedures, we will engage providers in chart-stimulated recall before the implementation strategies are tested, in the midst of strategy testing, and afterwards.

#### Analyses

##### Acceptability and feasibility

We will analyze acceptability and feasibility scores on the Acceptability of Intervention Measure and Feasibility of Intervention Measures using descriptive statistics for primary care and specialty mental health separately, which will allow us to explore how means and standard deviations on these measures compare across sites. We will collect and summarize qualitative perceptions of the feasibility and acceptability of the strategies.

##### Chart-stimulated recall

Preliminary fidelity estimates will be exploratory given that we will not be powered to detect effects.

The outcomes of Aim 3 will be preliminary data on the feasibility and acceptability of the implementation strategies, as well as exploratory data on provider fidelity to SSAI EBPs.

## Discussion

This project has the potential to increase the use of suicide prevention EBPs in settings in which individuals with suicidal ideation and behavior are particularly likely to present. The dual-pronged approach of including primary care and specialty mental health settings will increase the potential reach and subsequent impact of this research. Importantly, this cross-setting work will produce both specific knowledge on how to implement SSAI EBPs within each setting and generalizable knowledge across settings. Thus, the present study has the potential to accelerate the pace at which SSAI EBPs are deployed in multiple healthcare settings.

This project has several key strengths. First, it applies insights, frameworks, and methods from BE, which to date have not been applied systematically within implementation science to improve suicide prevention [38]. Second, it utilizes innovative methods, including RCAs from industry to “fail fast and learn quickly” in the search for effective implementation strategies [57]. Third, it utilizes a multimethod approach to identify behavioral barriers that may impede implementation. Fourth, it will provide preliminary indications of the mechanisms (i.e., assumptions) through which implementation strategies improve the use of SSAI EBPs [35, 36]. Thus, testing assumptions in the RCAs will provide key information on why strategies did or did not work, thereby allowing us to identify specific targets for subsequent trials. Fifth, the proposed work will allow for the identification of core determinants across settings that may hinder or facilitate SSAI EBP implementation, ultimately leading to a widely applicable set of implementation strategies [34].

Nonetheless, there are several potential limitations to this study. In particular, as this is a trial focused on establishing feasibility and acceptability, the sample size will be small, thereby limiting statistical power. However, this research will be pivotal in paving the way for larger confirmatory trials of the novel implementation strategies developed in this study. Furthermore, the RCAs used in the current study will allow us to prototype implementation strategies, designed in collaboration with key stakeholders, to optimize their effectiveness before conducting expensive and lengthy trials. Additionally, although the use of multiple settings enhances the generalizability of our work, the extent to which findings will be applicable to healthcare systems in other areas of the United States as well as in other countries, or to settings beyond primary care and specialty mental health (e.g., specialty medical clinics, college counseling centers), is an empirical question to test in future studies. Given the study’s focus on provider perspectives and behavior, patients and families were not recruited. The inclusion of patients and families in future work on this topic will be critical for building upon research that has assessed patient and family perspectives to date [58, 59] and for ensuring SSAI procedures are acceptable to a range of stakeholders.

Overall, this study will yield essential information on strategies for maximizing the implementation of EBPs that can reduce the likelihood of deaths by suicide. This work will be a critical building block for improving the quality of care and clinical outcomes in primary care and specialty mental health. Findings will spur further research on ways to augment the delivery of SSAI EBPs across settings.

## Data Availability

Not applicable.

## Abbreviations

EBP: Evidence-based practice
SSAI: Suicide screening, assessment, and intervention
RCA: Rapid cycle approaches
EAST: Easy, Attractive, Social, and Timely
BE: Behavioral economics
ALACRITY: Advanced Laboratories for Accelerating the Reach and Impact of Treatments for Youth and Adults with Mental Illness
